# Impact of differences in adenoma and proximal serrated polyp detection rate on the long-term effectiveness of FIT-based colorectal cancer screening

**DOI:** 10.1186/s12885-018-4375-9

**Published:** 2018-04-25

**Authors:** Maxime E. S. Bronzwaer, Marjolein J. E. Greuter, Arne G. C. Bleijenberg, Joep E. G. IJspeert, Evelien Dekker, Veerle M. H. Coupé

**Affiliations:** 1 Department of Gastroenterology and Hepatology, Academic Medical Center, University of Amsterdam, Meibergdreef 9, 1105 AZ Amsterdam, The Netherlands; 2 Department of Epidemiology and Biostatistics, VU University Medical Center, Amsterdam, The Netherlands

**Keywords:** Colorectal cancer, Screening, Health economic modeling, Adenoma detection rate, Proximal serrated polyp detection rate

## Abstract

**Background:**

Both the adenoma detection rate (ADR) and proximal serrated polyp detection rate (PSPDR) vary among endoscopists. It is unclear how these variations influence colorectal cancer (CRC) screening effectiveness. We evaluated the effect of variation in these detection rates on the long-term impact of fecal immunochemical test (FIT) based screening.

**Methods:**

The Adenoma and Serrated pathway to Colorectal CAncer (ASCCA) model was set up to simulate the Dutch national biennial FIT-based CRC screening program between 2014 and 2044. Adherence to FIT and colonoscopy was 73 and 92%. Besides a ‘no screening scenario’, several screening scenarios varying in ADR and PSPDR were evaluated. Using the available literature on colonoscopy miss rates led to a base-case ADR of 59% and PSPDR of 11%, which were varied with intervals of 3 and 2%.

**Results:**

Compared to no screening, FIT-screening in the base-case scenario reduced long-term mortality with 51.8%. At a fixed PSPDR of 11%, an increase in ADR from 44 to 62% would result in a 10.7% difference in mortality reduction. Using a fixed ADR of 59%, changing the PSPDR from 3 to 15% did not substantially influence long-term mortality (51.0 to 52.3%).

**Conclusions:**

An increase in ADR gradually reduces CRC burden in a FIT-based screening program, whereas an increase in PSPDR only minimally influences long-term outcomes at a population-level. The limited effect of the PSPDR can be explained by the limited sensitivity of FIT for serrated polyps (SPs). Other triage modalities aiming to detect relevant SPs should be explored.

## Background

Colorectal cancer (CRC) is one of the most prevalent causes of cancer-related morbidity and mortality in Western countries [1]. Both can be reduced by the detection of cancers at early, curable stages and by the detection and removal of colorectal adenomas, the most important CRC precursor lesions [2, 3]. Colonoscopy is the reference standard for the detection and removal of adenomas and its associated CRC mortality reduction is why CRC screening is implemented in many Western countries [2–4]. CRC screening programs can be divided in primary colonoscopy screening programs in which all participants undergo a screening colonoscopy, and screening programs in which the screening colonoscopy is preceded by a triage modality, such as non-invasive stool tests [4]. Only test-positives will undergo colonoscopy. The effectiveness of all CRC screening programs therefore relies on the quality of the colonoscopy, of which the adenoma detection rate (ADR) is the most established quality indicator [5–8]. In primary screening colonoscopy cohorts lower ADRs were associated with higher post-colonoscopy CRC and CRC mortality risks [5, 6].

An increasing body of evidence suggests that serrated polyps (SPs) also contribute to CRC oncogenesis [9–11]. Of all post-colonoscopy CRCs, a significant proportion seems to arise from proximal located SPs, presumably because of high lesion miss rates [12, 13]. As such, the detection of proximal SPs is of importance and the proximal serrated polyp detection rate (PSDPR) has been proposed as a screening colonoscopy quality indicator as well [14–17]. However, the PSPDR is not an established quality indicator, as the association between the PSDPR and the occurrence of post-colonoscopy CRCs has not been established yet [14, 17].

Both the ADR and the PSPDR are known to vary among endoscopists [5, 6, 14, 17–23]. Nonetheless, little is known about the effect of these variations in ADR and PSPDR on the effectiveness of a screening program using biennial fecal immunochemical testing (FIT) as a triage modality. Therefore, this study aimed to evaluate the effect of variation in ADR and PSPDR on the long-term impact of a biennial FIT-based CRC screening program using the Adenoma and Serrated pathway to Colorectal CAncer (ASCCA) model.

## Methods

### ASCCA model

The ASCCA model, which is extensively described elsewhere, was used for all analyses [24]. In brief, the natural history model incorporates two pathways to CRC: the adenoma-carcinoma pathway and the serrated pathway. The serrated pathway is assumed to contribute to 15% of CRC cases [25]. Individual health trajectories are simulated from age 20 to age 90 or death, whichever comes first. During their life, individuals can develop up to 10 adenomas and 10 SPs. In the model only hyperplastic polyps (HPs) and sessile serrated lesions (SSLs) were included as traditional serrated adenomas are very rare [26]. The development of each lesion in terms of growth in size is modelled independently. For adenomas, also the development of high-grade dysplasia and villosity is taken into account. Only advanced adenomas and SSLs can progress to CRC. Once an asymptomatic tumor has developed, there is an annual chance that the tumor becomes detected by symptoms or progresses to a more advanced stage. Table 3 in Appendix provides an overview of the natural history parameters. The model satisfactorily replicates Dutch colorectal lesion prevalence, CRC incidence and CRC mortality in the absence of screening [27, 28]. The natural history model is supplemented with a flexible screening and surveillance component, which can be set up to evaluate a range of screening and surveillance strategies. Parameters of the screening and surveillance component are updated regularly using the results of the national monitor of the Dutch CRC screening program [29].

### Dutch screening program and surveillance guidelines

The ASCCA model was set up to simulate the Dutch national CRC screening program; model parameters are shown in Table 1. The Dutch screening program was implemented in 2014 and involves biennial FIT-screening [30]. The implementation is phased; each year new birth cohorts are invited until the program is fully implemented in 2019. From 2019 onwards, all individuals aged 55 to 75 will be invited biennially. Individuals with a positive test outcome (cut-off 75 ng/ml) are referred for colonoscopy. FIT characteristics for detecting adenomas were obtained following a previously described calibration procedure [24]. We calibrated against the positivity rate, detection rates and positive predictive values of a Dutch screening pilot study [31]. For SPs, the positivity rate was assumed to be equal to one minus the specificity [32]. We assumed that during colonoscopy, all detected lesions are completely removed, with the exception of small HPs (< 5 mm) located in the rectosigmoid [33]. Adherence rates to FIT and FIT-positive colonoscopy were set at 73 and 92% based on the national monitor of the Dutch CRC screening program [29, 34].

**Table 1:** Overview of important model parameters

Variable	Base-case analysis		Sensitivity analysis		Reference
FIT-screening					National monitor of the Dutch CRC screening program [29, 34]
Participation FIT	0.73				
Adherence to FIT-positive colonoscopy	0.92				
Adherence to surveillance colonoscopy	0.92				
Primary colonoscopy screening					[27, 29]
Adherence to screening colonoscopy			0.22		
Adherence to surveillance colonoscopy			0.92		
FIT positivity rate per lesion	Men	Women	Men	Women	[31]
Healthy	0.96^a^	0.97^a^			
Diminutive adenoma	0.004	0.003			
Small adenoma	0.12	0.10			
Large adenoma	0.30	0.28			
Small SP	0.004	0.003	0.06	0.05	
Large SP	0.004	0.003	0.30	0.28	
Early stage CRC	0.50	0.50			
Late stage CRC	0.85	0.85			
Contribution of serrated pathway to CRC incidence	15%		30%		[12]
Complications after colonoscopy	0.0028				[35–37]
Fatal complications after colonoscopy	0.0001				[35–37]

FIT, fecal immunochemical test

^a^Specificity per individual

Colonoscopy surveillance is modelled in accordance with Dutch guidelines, which is guided by a risk score based on the number, size and location of the encountered colorectal polyps [33]. This risk score determines the surveillance interval, i.e. 3 or 5 years. If during FIT-positive colonoscopy no adenomas or only one small (≤ 1 cm) tubular adenoma is detected, the individual returns to screening after 10 years. Adherence to surveillance colonoscopy was assumed to be equal to that of FIT-positive colonoscopy, i.e. 92%, and surveillance ends at age 75.

### Detection settings

Besides the no screening comparator, we considered FIT-screening with different detection settings (varying both ADR and PSPDR). To estimate the ADR and PSPDR, the model was set up to simulate one round of FIT-screening (cut-off 75 ng/ml) in previously unscreened, asymptomatic individuals aged 55–75 years. First, we assumed size-specific detection rates per adenoma during FIT-positive colonoscopy as reported in a systematic review on adenoma miss rates to calculate the base-case ADR [7]. For SPs, lesion miss rates are not described in the literature. Since the flat appearance, proximal location and pale color of SPs hampers detection, a 10% lower detection rate per SP than per adenoma was assumed to calculate the base-case PSPDR [35]. Subsequently, the detection rate per adenoma was calibrated, such that the ADR increased and decreased with steps of 3% with a minimal ADR of 44%. As the prevalence of proximal SPs is lower than the adenoma prevalence, the PSPDR was increased and decreased with steps of 2% when calibrating the SP detection rate. A minimal PSPDR of 3% was assumed. The maximum ADR and PSPDR were reached under the assumption that all adenomas or SPs were detected. To achieve a specific ADR or PSPDR, the detection rates for the different size categories per lesion were varied jointly rather than individually. More specifically, we assumed that the absolute difference in detection rates between the different size categories per lesion type remained equal to those reported by Van Rijn et al. [7].

### Analyses and study outcomes

Screening was modelled from the introduction of the program in 2014 to 2044, while accounting for the phased rollout. We started with a population based on the 2013 Dutch population age-composition and assumed that this population will age in accordance with the predictions of the Dutch Central Bureau of Statistics [36].

For each FIT-screening scenario with different detection settings, yearly CRC incidence and mortality rates per 100,000 individuals and colonoscopy demand were evaluated. The FIT-screening scenario assuming the base-case ADR and PSPDR was compared to no screening. Subsequently, we assessed the impact of increasing the PSPDR with the ADR fixed at the base-case value as well as the impact of increasing the ADR with the PSPDR fixed at the base-case value.

### Sensitivity analyses

To assess the robustness of our results, we conducted one-way sensitivity analyses, i.e. varying only one parameter at the time. As there is much debate regarding the contribution of the serrated pathway to the CRC incidence [9–11], all FIT-screening scenarios with different detection settings were repeated assuming that 30% of CRCs arise from SPs instead of 15% used in the base-case analyses. Furthermore, we assumed that FIT detects adenomas and SPs equally well (Table 1).

In order to evaluate the impact of surveillance colonoscopy on the study outcomes, we repeated all analyses assuming an alternative strategy of FIT screening without surveillance, in which individuals considered at intermediate or high risk for metachronous lesions at FIT-positive colonoscopy return to FIT-screening after 2 years. Those at low risk return to the screening program after 10 years [37]. To allow for comparability of model results with other studies on ADR variances, all analyses were repeated assuming a fully implemented primary colonoscopy screening program. In this program, individuals aged 55 to 75 are invited every 10 years to undergo screening colonoscopy and dependent on the findings, may enter colonoscopy surveillance. Adherence rates for screening and surveillance colonoscopy were set at 22 and 92% [27, 29]. To evaluate the maximal impact of changes in ADR and PSPDR, also primary colonoscopy screening assuming perfect compliance was simulated.

## Results

### Adenoma and proxsimal SP detection rates

Table 2 shows the results of calibrating the ADR and PSPDR in one round of FIT-screening in previously unscreened individuals. Assuming detection rates per adenoma based on Van Rijn et al. led to an ADR of 59% [7]. This was considered the base-case ADR. The maximal ADR of 62% was reached when assuming that all adenomas were detected during FIT-positive colonoscopy. A minimal ADR of 44% was assumed for which the detection rates of diminutive, small and large adenomas were 36, 49 and 60%. Thus, the plausible ADR range is between 44 and 62%.

**Table 2:** ADR and PSPDR in one round of screening in previously unscreened individuals aged 55–75 years^a^

Calibrated detection rate per adenoma		ADR in previously unscreened individuals aged 55–75 years undergoing one round of		Calibrated detection rate per SP	PSPDR in previously unscreened individuals aged 55–75 years undergoing one round of	
		FIT- screening	Primary colonoscopy screening		FIT- screening	Primary colonoscopy screening
< 6 mm:	36%					
6–9 mm:	49%	44%	21%	< 10 mm: 15%	3%	3%
≥ 10 mm:	60%			≥ 10 mm: 33%		
< 6 mm:	42%					
6–9 mm:	55%	47%	23%	< 10 mm: 27%	5%	5%
≥ 10 mm:	66%			≥ 10 mm: 45%		
< 6 mm:	49%					
6–9 mm:	62%	50%	26%	< 10 mm: 40%	7%	8%
≥ 10 mm:	73%			≥ 10 mm: 58%		
< 6 mm:	56%					
6–9 mm:	69%	53%	28%	< 10 mm: 54%	9%	10%
≥ 10 mm:	80%			≥ 10 mm: 72%		
< 6 mm:	65%					
6–9 mm:	78%	56%	30%	< 10 mm: 68%	11%^b^[8]	12%
≥ 10 mm:	89%			≥ 10 mm: 86%		
< 6 mm:	74%					
6–9 mm:	87%	59%^b^[8]	33%	< 10 mm: 85%	13%	14%
≥ 10 mm:	98%			≥ 10 mm: 100%		
< 6 mm:	100%					
6–9 mm:	100%	62%	37%	< 10 mm: 100%	15%	15%
≥ 10 mm:	100%			≥ 10 mm: 100%		

^a^27% of individuals aged 55–59, 25% of individuals aged 60–64, 24% of individuals aged 65–69 and 25% of individuals aged 70–75

^b^an ADR of 59% and a PSPDR of 11% were considered as the base-case detection setting

For SPs, 10% lower detection rates per SP compared to the detection rates per adenoma were assumed, leading to a base-case PSPDR of 11% [7]. Assuming that all SPs are detected during FIT-positive colonoscopy led to a maximal PSPDR of 15%. We assumed a minimal PSPDR of 3% for which the detection rates were 15 and 33% for small and large SPs. Therefore, the plausible range for the PSPDR is between 3 and 15%. Table 2 also reports ADRs and PSPDRs for one round of primary colonoscopy screening.

### CRC burden and colonoscopy demand

**Fig. 1:**
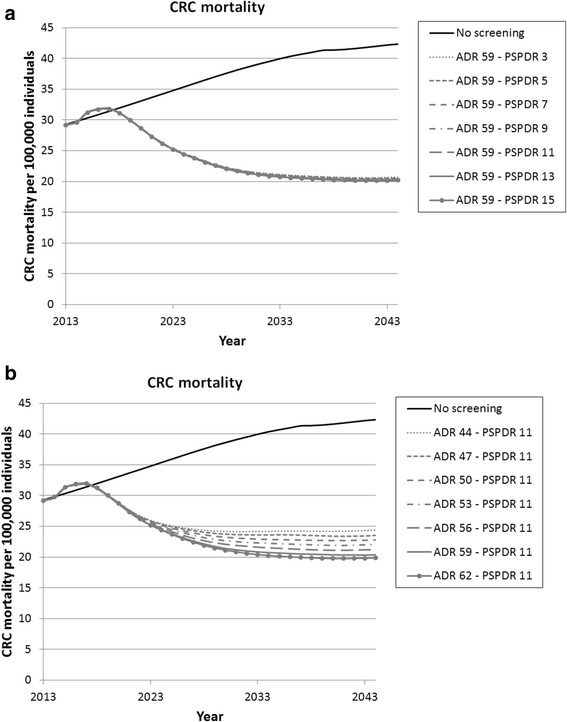
Long-term reduction in CRC mortality due to FIT-screening for different PSPDRs at a fixed ADR of 59% (**a**) and different ADRs at a fixed PSPDR of 11% (**b**)

In 2013, CRC incidence and mortality rates were 74.0 cases and 29.3 deaths per 100,000 individuals. In the absence of screening, CRC incidence and mortality are predicted to increase to 104.3 and 42.3 per 100,000 individuals in 2044 due to aging of the population. In the base-case detection setting, 30 years of FIT-screening led to a 36.7% reduction in CRC incidence and a 51.8% reduction in CRC mortality compared to no screening. When the ADR was fixed at 59% and a PSPDR of 3% was assumed, CRC mortality reduction was 51.0% compared to no screening (Fig. 1). This reduction increased with 1.3 to 52.3% when the PSPDR was increased to 15%. At a fixed PSPDR of 11% and when an ADR of 44% was assumed, CRC mortality reduction was 42.4% compared to no screening. Increasing the ADR to 62% led to a model-predicted mortality reduction of 53.1%, i.e. an increase of 10.7%. Similar patterns were observed for the CRC incidence reduction as shown in Fig. 3 in Appendix.

In the base-case detection setting 120,862 colonoscopies are required in 2044. Changes in the PSPDR at a fixed ADR of 59% did not influence colonoscopy demand. On the other hand, when the ADR was increased from 44 to 62% at a fixed PSPDR of 11%, colonoscopy demand was predicted to differ with 21,726 colonoscopies per year in 2044.

### Sensitivity analyses

**Fig. 2:**
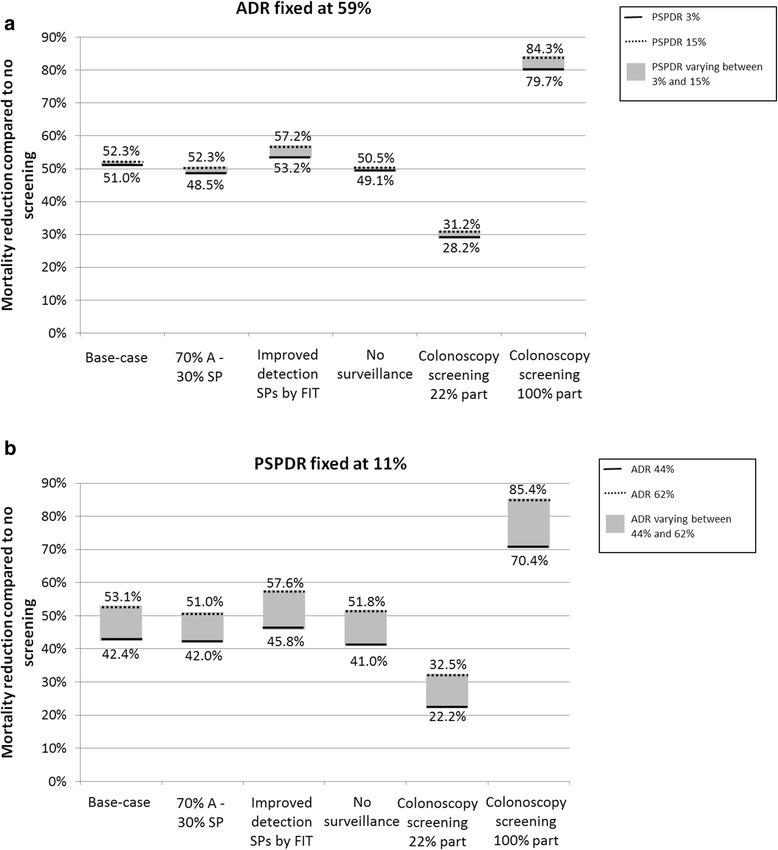
CRC mortality reduction compared to no screening for the base-case analysis and sensitivity analyses with the PSPDR varying between 3 and 15% at a fixed ADR of 59% (**a**) and with the ADR varying between 44 and 62% at a fixed PSPDR of 11% (**b**)

**Fig. 3:**
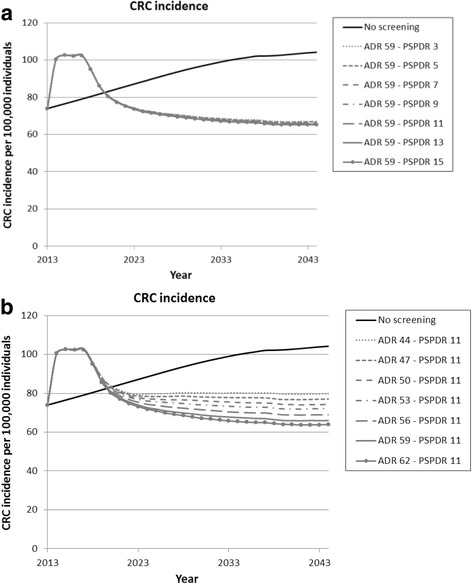
Long-term reduction in CRC incidence due to FIT-screening for different PSPDRs at a fixed ADR of 59% (**a**) and different ADRs at a fixed PSPDR of 11% (**b**)

Under the assumption that 30% of all CRCs develop according to the serrated pathway, the difference in mortality reduction when increasing the PSPDR over its plausible range (from 3 to 15%) at a fixed ADR of 59% was slightly larger than in the base-case analysis, with an increase of 2.1% from 48.5 to 50.6% (Fig. 2). The impact of increasing the PSPDR became more pronounced under the assumption that FIT has comparable sensitivity for adenomas and SPs; the difference in mortality reduction when increasing the PSPDR over its plausible range was 3.9% (from 53.2 to 57.2%). When considering a fixed PSPDR and variable ADR (plausible range from 44 to 62%), changes in contribution of the serrated pathway and detection of SPs by FIT led to a slightly smaller and a slightly greater difference in mortality reduction compared to the base-case analysis.

Evaluating the alternative strategy of FIT screening without surveillance, in which all individuals who were considered at intermediate or high risk at FIT-positive colonoscopy returned to screening after 2 years, we found comparable patterns with the base-case analysis. The difference in mortality reduction was 1.4% (increased from 49.1 to 50.5%) when the PSPDR was increased over its plausible range at a fixed ADR of 59%. When the PSPDR was fixed and the ADR was increased over its plausible range, the difference in mortality reduction increased with 10.8% (from 41.0 to 51.8%).

**Fig. 4:**
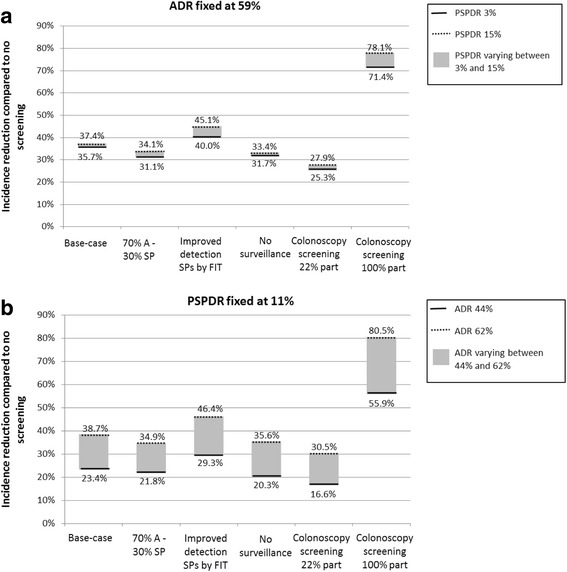
CRC incidence reduction compared to no screening for the base-case analysis and sensitivity analyses with the PSPDR varying between 3 and 15% at a fixed ADR of 59% (**a**) and with the ADR varying between 44 and 62% at a fixed PSPDR of 11% (**b**)

Also when the analyses were repeated assuming a fully implemented primary colonoscopy screening program with 22% colonoscopy adherence, similar patterns were observed [27]. An increase in the PSPDR over the plausible range only slightly increased the mortality reduction (from 28.2 to 31.2%), whereas an increase in ADR over its plausible range led to a considerable higher mortality reduction (from 22.2 to 32.5%). The maximal impact of an increase in detection rates was evaluated by assuming colonoscopy screening with perfect compliance. When the ADR was fixed, the difference in mortality reduction when increasing the PSPDR over its plausible range was 4.6% (from 79.7 to 84.3%). When the PSPDR was fixed, an increase in ADR over the plausible range led to a 15% difference in mortality reduction (from 70.4 to 85.4%). Results for CRC incidence were similar as shown in Fig. 4 in Appendix.

## Discussion

Based on the ASCCA model, an increase in ADR will gradually reduce CRC incidence and mortality in a biennial FIT-based CRC screening program, whereas an increase of the PSPDR does only minimally impact CRC burden at a population-level. Similar results were found when an alternative strategy of FIT screening without surveillance was evaluated. The impact of an increased PSPDR on long term-outcomes only slightly increased when assuming a 30% instead of 15% contribution of the serrated pathway and under the assumption that FIT would have a comparable sensitivity for adenomas and SPs. The maximum impact of changing either the PSPDR (from 3 to 15%) or ADR (from 44 to 62%) on mortality reduction due to screening was observed when a colonoscopy screening programme with perfect compliance was modelled. In that case, mortality reductions varied with 4.6 and 15% when varying the PSPDR and ADR over its plausible range.

There are two explanations for the limited influence of an increased PSPDR on the model-predicted effectiveness of FIT-based screening. Firstly, only 15–30% of all CRCs originate from the serrated pathway [11]. When assuming a 30% contribution of the serrated pathway to CRC incidence, CRC mortality reduction due to screening varied with 3.8% when increasing the PSPDR over its plausible range compared to 1.3% in the base-case scenario wherein a 15% contribution was assumed. Secondly, under the assumption that FIT has a comparable sensitivity for both adenomas and SPs, a 4.0% difference in mortality reduction by increasing the PSPDR over its plausible range was found. FIT is known to be ineffective to detect clinically relevant SPs, such as larger and/or proximally located HPs and SSLs, since these lesions seldom bleed [9, 11, 38, 39]. This is also supported by our calibration analysis in which equal detection rates per SP led to similar PSPDRs for FIT-screening and colonoscopy screening. In other words, FIT-screening does not lead to a subgroup of individuals referred for colonoscopy that has an increased SP prevalence. Contrastingly, the ADR was considerably higher after preselection with FIT compared to colonoscopy screening when assuming equal detection rates per adenoma. Positivity of FIT in individuals having relevant SPs is most likely due to the frequent co-occurrence of synchronous advanced adenomas or CRC [40].

The majority of individuals harbouring relevant SPs without concurrent adenomas will therefore not benefit from FIT-based screening. Particularly these individuals are at risk of developing a FIT interval cancer, as it is suggested that SPs, once dysplastic may evolve relatively quickly into malignancy [41]. Improved detection of proximal SPs during colonoscopy is only effective for improving the effectiveness of a CRC screening program, if colonoscopy is used as a primary screening modality or when a triage test would preselect individuals at increased risk for relevant SPs as well as for advanced adenomas and CRC. Molecular stool testing has shown promising results. However, costs, test specificity, and ease to perform should improve to become a realistic alternative to FIT [32]. Currently, whole stool samples are needed to enable molecular testing. This could be burdensome for screenees and will influence adherence rates, which is crucial for population-based screening programs [32].

Irrespective of the used triage modality, colonoscopy will remain the reference standard to detect and resect adenomas, SPs and cancer. To ensure the effectiveness of a screening program, quality assurance and monitoring the quality of colonoscopy is of paramount importance. To obtain and assure high quality within the Dutch national CRC screening program, national requirements were set for professionals performing screening colonoscopies. Only endoscopists satisfying pre-defined quality requirements are accredited to perform screening colonoscopies. During the accreditation process, the knowledge and skills of endoscopists are tested by an e-learning, by measuring evidence-based quality indicators and by evaluating the practical skills during colonoscopy [42].

The ADR is endorsed as the most important (screening) colonoscopy quality indicator, since it is inversely correlated with the occurrence of post-colonoscopy CRCs cancers and CRC mortality in large primary screening colonoscopy cohorts [5, 6]. However, ADR is criticized as being slightly imprecise, as it does not provide information about incremental adenomas detected besides the first, resulting in the ‘one and done phenomenon’[43]. Ideally, reporting of the ADR would be combined with a quality indicator reporting on the total number of detected adenomas [43]. In contrast to these data on ADR, no prospective studies evaluating the association between the PSPDR and the risk of interval cancers have been performed and recommendations for PSPDR thresholds are yet to be determined [14, 17]. As a consequence it can be hypothesized that the ‘one and done phenomenon’ currently does not apply to the PSPDR. Furthermore, both ADR and PSPDR do not select for neoplastic lesions having a higher neoplastic potential. The histopathological subtyping of SPs tends to be difficult, resulting in a broad diagnostic variability between pathologists [44]. However, by choosing the total group of SP located in the proximal colon, this interobserver variability among pathologists should not influence the results.

Both ADR and PSPDR vary widely, suggesting important lesion miss rates in low detecting endoscopists [5, 6, 14, 17–23]. Up to date, no studies have assessed interventions to improve the PSPDR. In contrast, several strategies aimed to improve ADR, including simple feedback, involvement of endoscopy nurses and mandating longer colonoscope withdrawal times, as well as multifaceted strategies involving education, audit and feedback. However, all methods had limited effect on ADR [45–49]. The minor impact and poor performance of most interventions may be caused by the paucity of evidence on appropriate factors to target for modification [50].

The interpretation of detection rates is difficult. This is due to the fact that besides endoscopy skills, detection rates are also influenced by the primary screening test and by the characteristics of the screening population, such as age, gender, screening history and prevalence of neoplastic lesions [18]. Thus, detection rates can only be interpreted in the context of the same screening setting. The calibrated detection rates in this study are based on one round of FIT-screening in previously unscreened, asymptomatic individuals aged 55–75 years. It should be noted that this differs from the Dutch CRC screening program which includes a phased implementation. During the implementation phase, selective cohorts are invited for screening starting with primarily older cohorts.

To the best of our knowledge this is the first microsimulation study investigating the influence of both the ADR and the PSPDR on the effectiveness of a biennially FIT-based as well as a primary colonoscopy screening program. Three other microsimulation studies estimated the effectiveness of primary colonoscopy screening at different levels of adenoma detection, also showing that higher ADRs were associated with important CRC incidence and mortality reductions [51–53]. The study by Meester et al. also investigated the effectiveness of annual FIT-based screening, showing a higher CRC related mortality in lower ADR settings [53]. An important difference between these models and the ASCCA model is the fact that both the adenoma-carcinoma pathway and the serrated pathway are included in the ASCCA model, whereas the other models only incorporate the adenoma-carcinoma pathway. This enabled us to also evaluate the impact of improvements in the PSPDR on CRC incidence and mortality reductions.

However, important limitations have to be acknowledged as well. First, we assumed a 10% lower detection rates rated for SPs than for adenomas to estimate the base-case PSPDR. Currently, the exact miss rates of SPs remain to be determined. However it is possible that the actual miss rates of SPs are higher than assumed in our base-case analysis, caused by the flat appearance, proximal location and pale color of SPs hampering detection [35]. On the other hand, the adenoma miss rates of colonoscopies performed nowadays may potentially be lower than miss rates reported by Van Rijn et al [7]. Since the publication of this study, the awareness of high quality colonoscopy has increased, accompanied by important improvements in the colonoscopy equipment, such as the application of high-definition colonoscopes and advanced imaging techniques. However, recently no new back-to-back studies have been published. To account for the uncertainty regarding this parameter however, we have evaluated a range of miss rates for both adenomas and SPs.

## Conclusions

In conclusion, an increase in ADR gradually will reduce CRC incidence and mortality in a biennial FIT-based screening program after 30-years of follow-up, whereas an increase of the PSPDR does only minimally influence long-term outcomes on a population-level. This limited effect of the PSPDR is partly explained by our assumption of a 15% contribution of the serrated pathway to the development of CRC, but more importantly by the limited diagnostic accuracy of FIT for SPs. Other triage modalities aiming to detect advanced SPs should be further explored.

## Appendix

**Table 3:** Natural history parameters of the ASCCA model

Natural history parameters	One-year transition probabilities		References
Adenoma-carcinoma pathway			
Adenoma incidence men (No adenoma to diminutive adenoma)			(A1-A3)
Age 20–39	0.003		
Age 40–49	0.007		
Age 50–54	0.019		
Age 55–59	0.022		
Age 60–64	0.024		
Age 65–69	0.028		
Age 70–74	0.033		
Age 75–90	0.035		
Adenoma incidence women			(A1-A3)
Incidence factor	0.6^a^		
Personal risk index adenoma-carcinoma pathway			(A2,A3)
Standard deviation	1.6^a^		
Progression in size			(A1,A3-A5)
Diminutive to small adenoma	0.07		
Small to large adenoma	0.10		
Regression in size			(A1,A3)
Small to diminutive adenoma	0.25		
Large to small adenoma	0.15		
Dysplasia (Low grade to high grade)			(A1,A3)
Diminutive adenoma	0.004		
Small adenoma	0.009		
Large adenoma	0.010		
Villosity (Tubular to tubulovillous/villous)			(A1,A3)
Diminutive adenoma	0.004		
Small adenoma	0.025		
Large adenoma	0.085		
Progression from AA to CRC^b^	Shape	Scale	(A6)
Men	2^a^	29^a^	
Women	2^a^	27^a^	
Serrated pathway			
Serrated lesion incidence men (No serrated lesion to small serrated lesion)	SSA	HP	(A1,A3)
Age 20–25	0.0001	0.001	
Age 25–29	0.0001	0.001	
Age 30–34	0.0001	0.002	
Age 35–39	0.0001	0.004	
Age 40–44	0.0006	0.007	
Age 45–49	0.0015	0.010	
Age 50–54	0.0016	0.010	
Age 55–59	0.0014	0.006	
Age 60–64	0.0008	0.004	
Age 65–69	0.0008	0.004	
Age 70–74	0.0007	0.002	
Age 75–79	0.0006	0.002	
Age 80–84	0.0005	0.002	
Age 85–90	0.0004	0.002	
Serrated lesion incidence women			
Incidence factor SSA	0.7^a^		(A1,A3)
Incidence factor HP	0.7^a^		
Personal risk index serrated pathway			(A1,A3)
Standard deviation	1.7^a^		
Progression in size			(A1,A3)
Small to large serrated lesion	0.028		
Regression in size			(A1,A3)
Small HP to no serrated lesion	0.0		
Large HP to small HP	0.4		
Progression to CRC	0.006		(A6)
CRC			
CRC	Dwell time in years	Stage distribution detected CRC	(A7,A8)
Stage 1	2.5^a^	0.19^a^	
Stage 2	2.0^a^	0.31^a^	
Stage 3	1.5^a^	0.49^a^	
Stage 4	1.0^a^	0.01^a^	

^a^Parameter value instead of yearly transition probability

^b^Weibull distribution

## Abbreviations

ADR: Adenoma detection rate
ASSCA: Adenoma and Serrated pathway to Colorectal CAncer
CRC: Colorectal cancers
FIT: Fecal immunochemical test
HP: Hyperplastic polyp
PSPDR: Proximal serrated polyp detection rate
SP: Serrated polyp
SSL: Sessile serrated lesion

## References

[1] Torre LA, Bray F, Siegel RL, Ferlay J, Lortet-Tieulent J, Jemal A (2015). Global cancer statistics, 2012. CA Cancer J Clin.

[2] Zauber AG, Winawer SJ, O'Brien MJ, Lansdorp-Vogelaar I, van Ballegooijen M, Hankey BF (2012). Colonoscopic polypectomy and long-term prevention of colorectal-cancer deaths. N Engl J Med.

[3] Winawer SJ, Zauber AG, Ho MN, O'Brien MJ, Gottlieb LS, Sternberg SS (1993). Prevention of colorectal cancer by colonoscopic polypectomy. The National Polyp Study Workgroup. N Engl J Med.

[4] Schreuders EH, Ruco A, Rabeneck L, Schoen RE, Sung JJ, Young GP (2015). Colorectal cancer screening: a global overview of existing programmes. Gut.

[5] Kaminski MF, Regula J, Kraszewska E, Polkowski M, Wojciechowska U, Didkowska J (2010). Quality indicators for colonoscopy and the risk of interval cancer. N Engl J Med.

[6] Corley DA, Jensen CD, Marks AR, Zhao WK, Lee JK, Doubeni CA (2014). Adenoma detection rate and risk of colorectal cancer and death. N Engl J Med.

[7] van Rijn JC, Reitsma JB, Stoker J, Bossuyt PM, van Deventer SJ, Dekker E (2006). Polyp miss rate determined by tandem colonoscopy: a systematic review. Am J Gastroenterol.

[8] Robertson DJ, Lieberman DA, Winawer SJ, Ahnen DJ, Baron JA, Schatzkin A (2014). Colorectal cancers soon after colonoscopy: a pooled multicohort analysis. Gut.

[9] Rex DK, Ahnen DJ, Baron JA, Batts KP, Burke CA, Burt RW (2012). Serrated lesions of the colorectum: review and recommendations from an expert panel. Am J Gastroenterol.

[10] Jass JR (2007). Classification of colorectal cancer based on correlation of clinical, morphological and molecular features. Histopathology.

[11] Bettington M, Walker N, Clouston A, Brown I, Leggett B, Whitehall V (2013). The serrated pathway to colorectal carcinoma: current concepts and challenges. Histopathology.

[12] Arain MA, Sawhney M, Sheikh S, Anway R, Thyagarajan B, Bond JH (2010). CIMP status of interval colon cancers: another piece to the puzzle. Am J Gastroenterol.

[13] Nishihara R, Ogino S, Chan AT (2013). Colorectal-cancer incidence and mortality after screening. N Engl J Med.

[14] IJspeert JE, van Doorn SC, van der Brug YM, Bastiaansen BA, Fockens P, Dekker E (2015). The proximal serrated polyp detection rate is an easy-to-measure proxy for the detection rate of clinically relevant serrated polyps. Gastrointest Endosc.

[15] Kahi CJ, Hewett DG, Norton DL, Eckert GJ, Rex DK (2011). Prevalence and variable detection of proximal colon serrated polyps during screening colonoscopy. Clin Gastroenterol Hepatol.

[16] de Wijkerslooth TR, Stoop EM, Bossuyt PM, Tytgat KM, Dees J, Mathus-Vliegen EM (2013). Differences in proximal serrated polyp detection among endoscopists are associated with variability in withdrawal time. Gastrointest Endosc.

[17] Zorzi M, Senore C, Da Re F, Barca A, Bonelli LA, Cannizzaro R, et al. Detection rate and predictive factors of sessile serrated polyps in an organised colorectal cancer screening programme with immunochemical faecal occult blood test: the EQuIPE study (Evaluating Quality Indicators of the Performance of Endoscopy). Gut. 2017;66(7):1233–1240. 10.1136/gutjnl-2015-310587. Epub 2016 Feb 19. 10.1136/gutjnl-2015-310587 26896459

[18] Hilsden RJ, Bridges R, Dube C, McGregor SE, Naugler C, Rose SM (2016). Defining benchmarks for adenoma detection rate and adenomas per colonoscopy in patients undergoing colonoscopy due to a positive fecal immunochemical test. Am J Gastroenterol.

[19] de Jonge V, Sint Nicolaas J, Cahen DL, Moolenaar W, Ouwendijk RJ, Tang TJ (2012). Quality evaluation of colonoscopy reporting and colonoscopy performance in daily clinical practice. Gastrointest Endosc.

[20] Chen SC, Rex DK (2007). Endoscopist can be more powerful than age and male gender in predicting adenoma detection at colonoscopy. Am J Gastroenterol.

[21] Bretagne JF, Hamonic S, Piette C, Manfredi S, Leray E, Durand G (2010). Variations between endoscopists in rates of detection of colorectal neoplasia and their impact on a regional screening program based on colonoscopy after fecal occult blood testing. Gastrointest Endosc.

[22] Imperiale TF, Glowinski EA, Juliar BE, Azzouz F, Ransohoff DF (2009). Variation in polyp detection rates at screening colonoscopy. Gastrointest Endosc.

[23] IJspeert JE, de Wit K, van der Vlugt M, Bastiaansen BA, Fockens P, Dekker E (2016). Prevalence, distribution and risk of sessile serrated adenomas/polyps at a center with a high adenoma detection rate and experienced pathologists. Endoscopy.

[24] Greuter MJ, Xu XM, Lew JB, Dekker E, Kuipers EJ, Canfell K (2014). Modeling the adenoma and serrated pathway to colorectal CAncer (ASCCA). Risk Anal.

[25] Ijspeert JEG, Vermeulen L, Meijer GA, Dekker E (2015). Serrated neoplasia-role in colorectal carcinogenesis and clinical implications. Nat Rev Gastroenterol Hepatol.

[26] Burke CA, Snover DC (2012). Editorial: sessile serrated adenomas and their pit patterns: we must first see the forest through the trees. Am J Gastroenterol.

[27] Stoop EM, de Haan MC, de Wijkerslooth TR, Bossuyt PM, van Ballegooijen M, Nio CY (2012). Participation and yield of colonoscopy versus non-cathartic CT colonography in population-based screening for colorectal cancer: a randomised controlled trial. Lancet Oncol.

[28] IKNL. Dutch National Cancer Registry https://www.cijfersoverkanker.nl/.

[29] National Institute for Public Health and the Environment EM (2016). NKI/AvL National Monitor National Colorectal Cancer Screening Programme 2015.

[30] Council NH (2009). Bevolkingsonderzoek naar darmkanker.

[31] van Rossum LG, van Rijn AF, Laheij RJ, van Oijen MG, Fockens P, van Krieken HH (2008). Random comparison of guaiac and immunochemical fecal occult blood tests for colorectal cancer in a screening population. Gastroenterology.

[32] Imperiale TF, Ransohoff DF, Itzkowitz SH, Levin TR, Lavin P, Lidgard GP (2014). Multitarget stool DNA testing for colorectal-cancer screening. N Engl J Med.

[33] Dekker EvL, ME; Hazewinkel, Y; Sanduleanu, S; Vasen, HF; Lansdorp-Vogelaar I, et al. Nederlandse Richtlijn Coloscopie Surveillance. http://wwwmdlnl/uploads/240/1308/Richtlijn_Coloscopie_Surveillance_definitief_2013pdf. 2013.

[34] Toes-Zoutendijk E, van Leerdam ME, Dekker E, van Hees F, Penning C, Nagtegaal I, et al. Real-time monitoring of results during first year of Dutch colorectal Cancer screening program and optimization by altering fecal immunochemical test cut-off levels. Gastroenterology. 2017; 152(4):767–775.e2. 10.1053/j.gastro.2016.11.022. Epub 2016 Nov 24. 10.1053/j.gastro.2016.11.022 27890769

[35] Hazewinkel Y, Lopez-Ceron M, East JE, Rastogi A, Pellise M, Nakajima T (2013). Endoscopic features of sessile serrated adenomas: validation by international experts using high-resolution white-light endoscopy and narrow-band imaging. Gastrointest Endosc.

[36] Statistics CBf. Available from: https://www.cbs.nl/. 2014.

[37] Greuter MJE, de Klerk CM, Meijer GA, Dekker E, Coupe VMH (2017). Screening for colorectal Cancer with fecal immunochemical testing with and without Postpolypectomy surveillance colonoscopy: a cost-effectiveness analysis. Ann Intern Med.

[38] JE IJ, Bossuyt PM, Kuipers EJ, Stegeman I, de Wijkerslooth TR, Stoop EM (2016). Smoking status informs about the risk of advanced serrated polyps in a screening population. Endosc Int Open.

[39] Heigh RI, Yab TC, Taylor WR, Hussain FT, Smyrk TC, Mahoney DW (2014). Detection of colorectal serrated polyps by stool DNA testing: comparison with fecal immunochemical testing for occult blood (FIT). PLoS One.

[40] Hazewinkel Y, de Wijkerslooth TR, Stoop EM, Bossuyt PM, Biermann K, van de Vijver MJ (2014). Prevalence of serrated polyps and association with synchronous advanced neoplasia in screening colonoscopy. Endoscopy.

[41] Bettington M, Walker N, Rosty C, Brown I, Clouston A, McKeone D (2017). Clinicopathological and molecular features of sessile serrated adenomas with dysplasia or carcinoma. Gut.

[42] National Institute for Public Health and the Environment MoH, Welfare and Sport (2012). Protocol for the authorization and audting of colonoscopy centers and endoscopists National screening programme for bowel cancer, First version.

[43] Wang HS, Pisegna J, Modi R, Liang LJ, Atia M, Nguyen M (2013). Adenoma detection rate is necessary but insufficient for distinguishing high versus low endoscopist performance. Gastrointest Endosc.

[44] IJspeert JE, Madani A, Overbeek LI, Dekker E, Nagtegaal ID (2017). Implementation of an e-learning module improves consistency in the histopathological diagnosis of sessile serrated lesions within a nationwide population screening programme. Histopathology.

[45] Kaminski MF, Anderson J, Valori R, Kraszewska E, Rupinski M, Pachlewski J (2016). Leadership training to improve adenoma detection rate in screening colonoscopy: a randomised trial. Gut.

[46] Wallace MB, Crook JE, Thomas CS, Staggs E, Parker L, Rex DK (2017). Effect of an endoscopic quality improvement program on adenoma detection rates: a multicenter cluster-randomized controlled trial in a clinical practice setting (EQUIP-3). Gastrointest Endosc.

[47] Coe SG, Crook JE, Diehl NN, Wallace MB (2013). An endoscopic quality improvement program improves detection of colorectal adenomas. Am J Gastroenterol.

[48] Ussui V, Coe S, Rizk C, Crook JE, Diehl NN, Wallace MB (2015). Stability of increased adenoma detection at colonoscopy. Follow-up of an endoscopic quality improvement program-EQUIP-II. Am J Gastroenterol.

[49] Corley DA, Jensen CD, Marks AR (2011). Can we improve adenoma detection rates? A systematic review of intervention studies. Gastrointest Endosc.

[50] Atkins L, Hunkeler EM, Jensen CD, Michie S, Lee JK, Doubeni CA (2016). Factors influencing variation in physician adenoma detection rates: a theory-based approach for performance improvement. Gastrointest Endosc.

[51] Meester RG, Doubeni CA, Lansdorp-Vogelaar I, Jensen CD, van der Meulen MP, Levin TR (2015). Variation in adenoma detection rate and the lifetime benefits and cost of colorectal Cancer screening: a microsimulation model. JAMA.

[52] Hassan C, Rex DK, Zullo A, Kaminski MF (2015). Efficacy and cost-effectiveness of screening colonoscopy according to the adenoma detection rate. United European Gastroenterol J.

[53] Meester RGS, Doubeni CA, Zauber AG, van Ballegooijen M, Corley DA, Lansdorp-Vogelaar I. Impact of adenoma detection on the benefit of faecal testing vs. colonoscopy for colorectal cancer. Int J Cancer. 2017;141(11):2359–67. 10.1002/ijc.30933. Epub 2017 Aug 31. 10.1002/ijc.30933 PMC5890914 28815573

